# Vinclozolin: 3-(3,5-di­chloro­phen­yl)-5-ethenyl-5-methyl-1,3-oxazolidine-2,4-dione

**DOI:** 10.1107/S1600536814012781

**Published:** 2014-06-07

**Authors:** Seonghwa Cho, Jineun Kim, Sangjin Lee, Tae Ho Kim

**Affiliations:** a Department of Chemistry and Research Institute of Natural Sciences, Gyeongsang National University, Jinju 660-701, Republic of Korea

## Abstract

In the title compound, C_12_H_9_Cl_2_NO_3_, which is the fungicide vinclozolin, the dihedral angle between the oxazolidine ring mean plane [r.m.s. deviation = 0.029 Å] and the benzene ring is 77.55 (8)°. In the crystal, mol­ecules are linked *via* C—H⋯O hydrogen bonds, forming chains along [010]. The chains are linked by short Cl⋯Cl contacts [3.4439 (3) and 3.5798 (3) Å], resulting in a three-dimensional architecture.

## Related literature   

For information on the toxicity and fungicidal properties of the title compound, see: van Ravenzwaay *et al.* (2013 ▶); Pothuluri *et al.* (2000 ▶). For a related crystal structure, see: Merino *et al.* (2010 ▶).
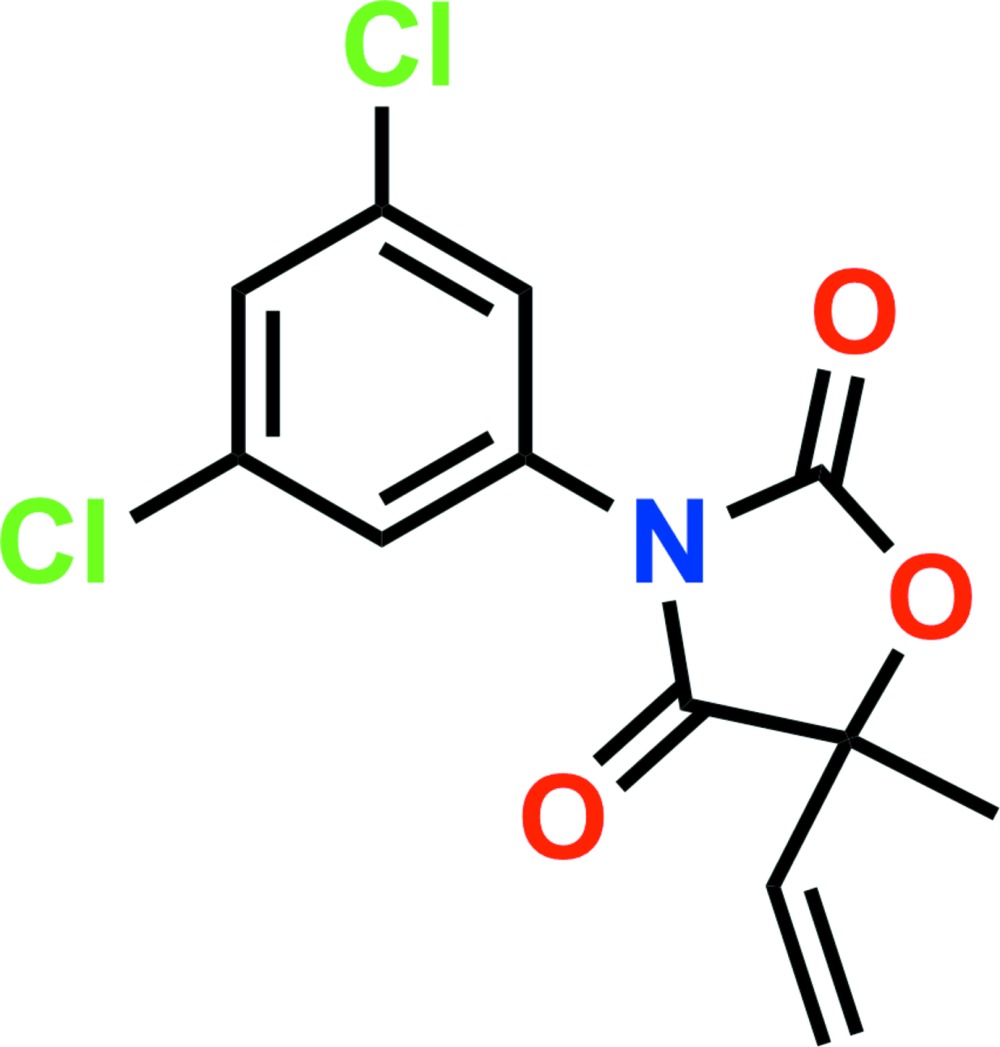


## Experimental   

### 

#### Crystal data  

C_12_H_9_Cl_2_NO_3_
*M* *_r_* = 286.10
Monoclinic, 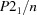

*a* = 15.0727 (12) Å
*b* = 5.2947 (5) Å
*c* = 15.5390 (12) Å
β = 100.916 (5)°
*V* = 1217.66 (18) Å^3^
*Z* = 4
Mo *K*α radiation
μ = 0.53 mm^−1^
*T* = 173 K
0.26 × 0.21 × 0.05 mm

#### Data collection  

Bruker APEXII CCD diffractometer
Absorption correction: multi-scan (*SADABS*; Bruker, 2009 ▶) *T*_min_ = 0.874, *T*_max_ = 0.974
7915 measured reflections
2103 independent reflections
1720 reflections with *I* > 2σ(*I*)
*R*_int_ = 0.030

#### Refinement  

*R*[*F* ^2^ > 2σ(*F* ^2^)] = 0.042
*wR*(*F* ^2^) = 0.115
*S* = 1.06
2103 reflections
163 parameters
H-atom parameters constrained
Δρ_max_ = 0.87 e Å^−3^
Δρ_min_ = −0.30 e Å^−3^

### 

Data collection: *APEX2* (Bruker, 2009 ▶); cell refinement: *SAINT* (Bruker, 2009 ▶); data reduction: *SAINT*; program(s) used to solve structure: *SHELXTL* (Sheldrick, 2008 ▶); program(s) used to refine structure: *SHELXTL*; molecular graphics: *DIAMOND* (Brandenburg, 2010 ▶); software used to prepare material for publication: *SHELXTL*.

## Supplementary Material

Crystal structure: contains datablock(s) global, I. DOI: 10.1107/S1600536814012781/sj5407sup1.cif

Structure factors: contains datablock(s) I. DOI: 10.1107/S1600536814012781/sj5407Isup2.hkl

Supporting information file. DOI: 10.1107/S1600536814012781/sj5407Isup3.cml

CCDC reference: 1006251

Additional supporting information:  crystallographic information; 3D view; checkCIF report

## Figures and Tables

**Table 1:** Hydrogen-bond geometry (Å, °)

*D*—H⋯*A*	*D*—H	H⋯*A*	*D*⋯*A*	*D*—H⋯*A*
C1—H1⋯O1^i^	0.95	2.59	3.454 (3)	151

Symmetry code: (i) 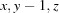.

**Fig. 1.**
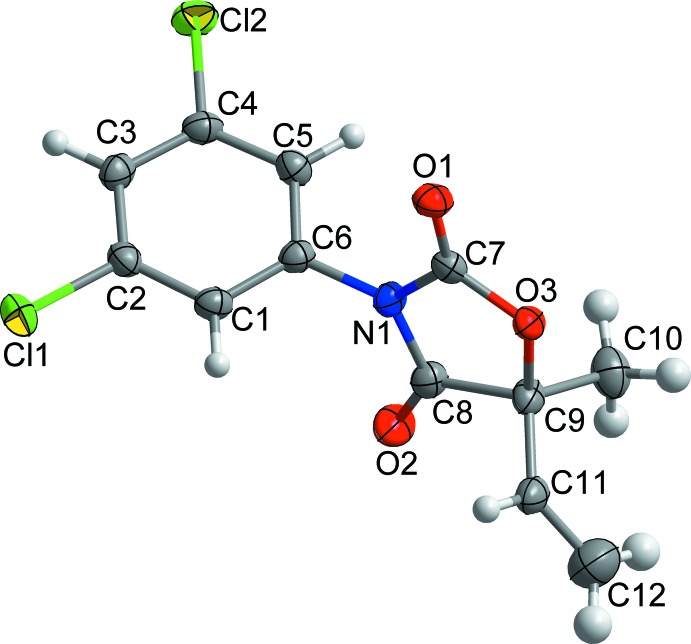
The molecular structure of the title compound. Displacement ellipsoids are drawn at the 50% probability level. H atoms are shown as small spheres of arbitrary radius.

**Fig. 2.**
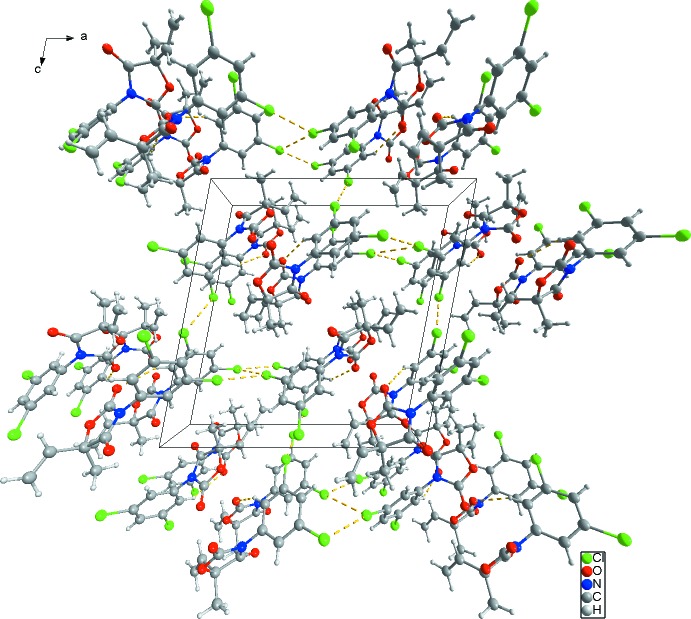
Crystal packing of the title compound with C—H···O hydrogen bonds and weak intermolecular Cl···Cl interactions shown as dashed lines.

## supplementary crystallographic information

### S1. Comment

The title compound vinclozolin, C_12_H_9_Cl_2_NO_3_, is a systemic dicarboximide fungicide used widely for the control of diseases in grapes, fruits, vegetables, ornamental plants, and turfgrass (Pothuluri *et al.*, 2000; van Ravenzwaay *et al.*, 2013), and its crystal structure is reported herein. In this compound (Fig. 1), the dihedral angle between the oxazolidine ring and the phenyl ring is 77.55 (8)°. All bond lengths and bond angles are normal and comparable to those observed in the crystal structure of a similar compound (Merino *et al.*, 2010).

In the crystal structure (Fig. 2), an intermolecular C—H···O hydrogen bond is observed (Table 1). In addition, short Cl···Cl contacts [Cl1···Cl1^ii^, 3.4439 (3) Å and Cl2···Cl2^iii^, 3.5798 (3) Å] are present. [symmetry codes: (ii), -*x*, *y*, -*z* + 1, and (iii), -*x* - 1/2, *y* - 1/2, -*z* + 1/2].

### S2. Experimental

The title compound was purchased from the Dr. Ehrenstorfer GmbH Company. Slow evaporation of a solution in CH_2_Cl_2_ gave single crystals suitable for X-ray analysis.

### S3. Refinement

All H-atoms were positioned geometrically and refined using a riding model with d(C—H) = 0.95 Å, *U*_iso_ = 1.2*U*_eq_(C) for aromatic C—H, d(C—H) = 0.95 Å, *U*_iso_ = 1.2*U*_eq_(C) for C*sp*^2^—H, and d(C—H) = 0.98 Å, *U*_iso_ = 1.5*U*_eq_(C) for CH_3_ groups.

### Crystal data

**Table:**

C_12_H_9_Cl_2_NO_3_	*F*(000) = 584
*M**_r_* = 286.10	*D*_x_ = 1.561 Mg m^−^^3^
Monoclinic, *P*2_1_/*n*	Mo *K*α radiation, λ = 0.71073 Å
Hall symbol: -P 2yn	Cell parameters from 4964 reflections
*a* = 15.0727 (12) Å	θ = 2.7–28.1°
*b* = 5.2947 (5) Å	µ = 0.53 mm^−^^1^
*c* = 15.5390 (12) Å	*T* = 173 K
β = 100.916 (5)°	Plate, colourless
*V* = 1217.66 (18) Å^3^	0.26 × 0.21 × 0.05 mm
*Z* = 4	

### Data collection

**Table:**

Bruker APEXII CCD diffractometer	2103 independent reflections
Radiation source: fine-focus sealed tube	1720 reflections with *I* > 2σ(*I*)
Graphite monochromator	*R*_int_ = 0.030
φ and ω scans	θ_max_ = 25.0°, θ_min_ = 1.7°
Absorption correction: multi-scan (*SADABS*; Bruker, 2009)	*h* = −15→17
*T*_min_ = 0.874, *T*_max_ = 0.974	*k* = −6→5
7915 measured reflections	*l* = −18→16

### Refinement

**Table:**

Refinement on *F*^2^	Primary atom site location: structure-invariant direct methods
Least-squares matrix: full	Secondary atom site location: difference Fourier map
*R*[*F*^2^ > 2σ(*F*^2^)] = 0.042	Hydrogen site location: inferred from neighbouring sites
*wR*(*F*^2^) = 0.115	H-atom parameters constrained
*S* = 1.06	*w* = 1/[σ^2^(*F*_o_^2^) + (0.0581*P*)^2^ + 0.9965*P*] where *P* = (*F*_o_^2^ + 2*F*_c_^2^)/3
2103 reflections	(Δ/σ)_max_ < 0.001
163 parameters	Δρ_max_ = 0.87 e Å^−^^3^
0 restraints	Δρ_min_ = −0.30 e Å^−^^3^

### Special details

**Table:**

Geometry. All e.s.d.'s (except the e.s.d. in the dihedral angle between two l.s. planes) are estimated using the full covariance matrix. The cell e.s.d.'s are taken into account individually in the estimation of e.s.d.'s in distances, angles and torsion angles; correlations between e.s.d.'s in cell parameters are only used when they are defined by crystal symmetry. An approximate (isotropic) treatment of cell e.s.d.'s is used for estimating e.s.d.'s involving l.s. planes.
Refinement. Refinement of *F*^2^ against ALL reflections. The weighted *R*-factor *wR* and goodness of fit *S* are based on *F*^2^, conventional *R*-factors *R* are based on *F*, with *F* set to zero for negative *F*^2^. The threshold expression of *F*^2^ > σ(*F*^2^) is used only for calculating *R*-factors(gt) *etc*. and is not relevant to the choice of reflections for refinement. *R*-factors based on *F*^2^ are statistically about twice as large as those based on *F*, and *R*- factors based on ALL data will be even larger.

### Fractional atomic coordinates and isotropic or equivalent isotropic displacement parameters (Å^2^)

**Table:**

	*x*	*y*	*z*	*U*_iso_*/*U*_eq_	
Cl1	0.01684 (5)	0.22311 (15)	0.42408 (5)	0.0385 (2)	
Cl2	−0.16975 (5)	0.99394 (15)	0.25170 (5)	0.0376 (2)	
O1	0.19649 (12)	0.9720 (4)	0.22124 (12)	0.0290 (5)	
O2	0.06758 (13)	0.2867 (4)	0.06863 (13)	0.0359 (5)	
O3	0.23229 (11)	0.7650 (3)	0.10675 (11)	0.0240 (4)	
N1	0.11223 (13)	0.6312 (4)	0.15799 (13)	0.0229 (5)	
C1	0.06127 (17)	0.4411 (5)	0.28171 (17)	0.0255 (6)	
H1	0.1090	0.3216	0.2872	0.031*	
C2	0.00147 (18)	0.4404 (5)	0.33904 (17)	0.0259 (6)	
C3	−0.06989 (17)	0.6085 (6)	0.33076 (17)	0.0274 (6)	
H3	−0.1110	0.6039	0.3702	0.033*	
C4	−0.07979 (17)	0.7832 (5)	0.26364 (17)	0.0271 (6)	
C5	−0.02013 (17)	0.7960 (5)	0.20586 (17)	0.0262 (6)	
H5	−0.0268	0.9199	0.1609	0.031*	
C6	0.04936 (16)	0.6214 (5)	0.21620 (16)	0.0233 (6)	
C7	0.18216 (17)	0.8082 (5)	0.16778 (16)	0.0224 (6)	
C8	0.11644 (17)	0.4631 (5)	0.09092 (17)	0.0259 (6)	
C9	0.19567 (18)	0.5543 (5)	0.04975 (17)	0.0258 (6)	
C10	0.1611 (2)	0.6600 (7)	−0.04096 (18)	0.0392 (8)	
H10A	0.1145	0.7872	−0.0380	0.059*	
H10B	0.1352	0.5231	−0.0803	0.059*	
H10C	0.2111	0.7381	−0.0633	0.059*	
C11	0.26703 (18)	0.3557 (6)	0.05662 (18)	0.0297 (7)	
H11	0.2954	0.3018	0.1135	0.036*	
C12	0.2928 (3)	0.2518 (8)	−0.0106 (2)	0.0583 (10)	
H12A	0.2657	0.3016	−0.0683	0.070*	
H12B	0.3386	0.1262	−0.0019	0.070*	

### Atomic displacement parameters (Å^2^)

**Table:**

	*U*^11^	*U*^22^	*U*^33^	*U*^12^	*U*^13^	*U*^23^
Cl1	0.0470 (5)	0.0333 (5)	0.0372 (4)	0.0032 (3)	0.0134 (3)	0.0124 (3)
Cl2	0.0288 (4)	0.0354 (5)	0.0501 (5)	0.0108 (3)	0.0113 (3)	0.0021 (3)
O1	0.0311 (10)	0.0223 (11)	0.0338 (10)	−0.0024 (8)	0.0068 (8)	−0.0051 (9)
O2	0.0336 (11)	0.0318 (13)	0.0446 (11)	−0.0144 (10)	0.0132 (9)	−0.0112 (10)
O3	0.0245 (9)	0.0209 (11)	0.0281 (9)	−0.0056 (8)	0.0090 (8)	−0.0028 (8)
N1	0.0200 (11)	0.0235 (13)	0.0260 (11)	−0.0028 (9)	0.0064 (9)	−0.0023 (9)
C1	0.0207 (13)	0.0237 (16)	0.0320 (14)	0.0007 (11)	0.0048 (11)	−0.0012 (12)
C2	0.0298 (14)	0.0213 (16)	0.0268 (13)	−0.0023 (12)	0.0056 (11)	0.0014 (11)
C3	0.0248 (14)	0.0273 (17)	0.0323 (14)	−0.0039 (12)	0.0109 (12)	−0.0039 (12)
C4	0.0219 (13)	0.0246 (16)	0.0349 (15)	0.0020 (11)	0.0055 (11)	−0.0032 (12)
C5	0.0256 (14)	0.0244 (16)	0.0284 (13)	−0.0020 (12)	0.0047 (11)	0.0026 (12)
C6	0.0209 (13)	0.0227 (15)	0.0271 (13)	−0.0012 (11)	0.0061 (11)	−0.0034 (11)
C7	0.0216 (13)	0.0190 (15)	0.0259 (13)	−0.0002 (11)	0.0023 (11)	0.0015 (12)
C8	0.0220 (13)	0.0269 (17)	0.0286 (14)	−0.0010 (12)	0.0044 (11)	−0.0016 (12)
C9	0.0286 (14)	0.0241 (16)	0.0265 (13)	−0.0092 (12)	0.0097 (11)	−0.0052 (11)
C10	0.0396 (17)	0.044 (2)	0.0324 (15)	−0.0095 (15)	0.0035 (13)	0.0032 (14)
C11	0.0310 (15)	0.0277 (17)	0.0342 (14)	−0.0048 (13)	0.0158 (12)	−0.0030 (12)
C12	0.060 (2)	0.058 (3)	0.062 (2)	0.006 (2)	0.0230 (19)	−0.006 (2)

### Geometric parameters (Å, º)

**Table:**

Cl1—C2	1.734 (3)	C3—H3	0.9500
Cl2—C4	1.739 (3)	C4—C5	1.388 (4)
O1—C7	1.192 (3)	C5—C6	1.383 (4)
O2—C8	1.199 (3)	C5—H5	0.9500
O3—C7	1.340 (3)	C8—C9	1.536 (4)
O3—C9	1.466 (3)	C9—C11	1.494 (4)
N1—C8	1.381 (3)	C9—C10	1.515 (4)
N1—C7	1.397 (3)	C10—H10A	0.9800
N1—C6	1.430 (3)	C10—H10B	0.9800
C1—C2	1.382 (4)	C10—H10C	0.9800
C1—C6	1.382 (4)	C11—C12	1.303 (4)
C1—H1	0.9500	C11—H11	0.9500
C2—C3	1.383 (4)	C12—H12A	0.9500
C3—C4	1.381 (4)	C12—H12B	0.9500
			
C7—O3—C9	111.06 (18)	O1—C7—N1	126.7 (2)
C8—N1—C7	111.9 (2)	O3—C7—N1	108.9 (2)
C8—N1—C6	125.8 (2)	O2—C8—N1	127.3 (2)
C7—N1—C6	122.2 (2)	O2—C8—C9	127.4 (2)
C2—C1—C6	117.9 (2)	N1—C8—C9	105.2 (2)
C2—C1—H1	121.1	O3—C9—C11	108.0 (2)
C6—C1—H1	121.1	O3—C9—C10	107.8 (2)
C1—C2—C3	121.9 (2)	C11—C9—C10	116.3 (2)
C1—C2—Cl1	118.9 (2)	O3—C9—C8	102.78 (19)
C3—C2—Cl1	119.2 (2)	C11—C9—C8	110.8 (2)
C4—C3—C2	118.2 (2)	C10—C9—C8	110.2 (2)
C4—C3—H3	120.9	C9—C10—H10A	109.5
C2—C3—H3	120.9	C9—C10—H10B	109.5
C3—C4—C5	122.1 (3)	H10A—C10—H10B	109.5
C3—C4—Cl2	118.8 (2)	C9—C10—H10C	109.5
C5—C4—Cl2	119.2 (2)	H10A—C10—H10C	109.5
C6—C5—C4	117.5 (3)	H10B—C10—H10C	109.5
C6—C5—H5	121.3	C12—C11—C9	124.0 (3)
C4—C5—H5	121.3	C12—C11—H11	118.0
C1—C6—C5	122.5 (2)	C9—C11—H11	118.0
C1—C6—N1	118.8 (2)	C11—C12—H12A	120.0
C5—C6—N1	118.7 (2)	C11—C12—H12B	120.0
O1—C7—O3	124.4 (2)	H12A—C12—H12B	120.0
			
C6—C1—C2—C3	1.8 (4)	C6—N1—C7—O1	−2.6 (4)
C6—C1—C2—Cl1	−177.9 (2)	C8—N1—C7—O3	1.4 (3)
C1—C2—C3—C4	−1.0 (4)	C6—N1—C7—O3	177.2 (2)
Cl1—C2—C3—C4	178.7 (2)	C7—N1—C8—O2	177.7 (3)
C2—C3—C4—C5	−0.8 (4)	C6—N1—C8—O2	2.1 (4)
C2—C3—C4—Cl2	179.4 (2)	C7—N1—C8—C9	−3.6 (3)
C3—C4—C5—C6	1.7 (4)	C6—N1—C8—C9	−179.2 (2)
Cl2—C4—C5—C6	−178.5 (2)	C7—O3—C9—C11	−120.7 (2)
C2—C1—C6—C5	−0.8 (4)	C7—O3—C9—C10	112.9 (2)
C2—C1—C6—N1	177.9 (2)	C7—O3—C9—C8	−3.6 (3)
C4—C5—C6—C1	−0.8 (4)	O2—C8—C9—O3	−177.1 (3)
C4—C5—C6—N1	−179.6 (2)	N1—C8—C9—O3	4.2 (3)
C8—N1—C6—C1	74.6 (3)	O2—C8—C9—C11	−61.9 (4)
C7—N1—C6—C1	−100.6 (3)	N1—C8—C9—C11	119.3 (2)
C8—N1—C6—C5	−106.7 (3)	O2—C8—C9—C10	68.3 (4)
C7—N1—C6—C5	78.2 (3)	N1—C8—C9—C10	−110.5 (3)
C9—O3—C7—O1	−178.6 (2)	O3—C9—C11—C12	−129.1 (3)
C9—O3—C7—N1	1.6 (3)	C10—C9—C11—C12	−7.9 (4)
C8—N1—C7—O1	−178.4 (3)	C8—C9—C11—C12	119.0 (3)

### Hydrogen-bond geometry (Å, º)

**Table:**

*D*—H···*A*	*D*—H	H···*A*	*D*···*A*	*D*—H···*A*
C1—H1···O1^i^	0.95	2.59	3.454 (3)	151

Symmetry code: (i) *x*, *y*−1, *z*.
